# Effect of Surgical Specimen Chain Management Information System on specimen management in operating room: a pilot Quasi-Experimental study

**DOI:** 10.1186/s12912-025-04288-y

**Published:** 2026-01-06

**Authors:** Xu Zhang, Xiaofen Yu, Mengtian Wang, Feiyan Shen, Jing Shen, Chunju Yang, Min Hong

**Affiliations:** Department of the Operating Room, Zhejiang Provincial People’s Hospital, Affiliated People’s Hospital, Hangzhou Medical College, Hangzhou, Zhejiang China

**Keywords:** Specimen management, Information system, Management principles, Specimen errors

## Abstract

**Background:**

The proper handling of surgical specimen during the pre-analytical phase directly impacts diagnostic accuracy and patient’s treatment plan. Specimen handling involves multiple steps and departments, making it prone to errors. Ensuring seamless operation at each stage and continuously reducing the incidence of specimen handling errors remain critical priorities for perioperative staff. Current research primarily focuses on improving management practice or enhancing equipment automation, with rare studies exploring the integration of management principles into information system.

**Methods:**

This research adopted a pilot Quasi-Experimental study, reporting followed the TREND statement guidelines. By convenience sampling, 1,780 surgical specimens collected from March to April 2025 (1,259 permanent specimens and 521 intraoperative frozen specimens) were assigned to the control group, while 1744 surgical specimens collected from May to June 2025 (1,259 permanent specimens and 485 intraoperative frozen specimens) comprised the intervention group. Control group utilized the existing Hospital Information System, whereas the intervention group employed the Surgical Specimen Chain Management Information System. Chi-square test and Mann-Whitney U test were conducted to compare specimen handling qualified rates and specimen handling duration between the two groups. Wilcoxon signed-rank test was employed to analyze the satisfaction of the operating room nurses.

**Results:**

After the implementation of the Surgical Specimen Chain Management Information System, the permanent specimen of intervention group exhibited a statistically significant improvement in qualified rate of specimen handling comparing with the control group in terms of fixative adequacy (*p* = 0.006, OR = 3.371, 95% CI:1.349–8.422), qualification of orientation(*P* = 0.045, OR = 3.019, 95% CI: 0.971–9.387), and total qualified specimen handling (*P* < 0.001, OR = 3.799, 95% CI: 2.048–7.047), with statistically significant differences and moderate to large effect sizes except the qualification of orientation. There was no statistically significant difference in the qualified rate of intraoperative frozen specimen handling between two groups. The duration of specimen handling of permanent specimen (*P* < 0.001, *R* = 0.59, 95% CI: 0.56–0.62) and intraoperative frozen specimen (*P* < 0.001, *R* = 0.47, 95% CI: 0.41–0.52) in the intervention group were significantly shorter than those in the control group, and the differences were statistically significant along with a medium to large effect sizes. Additionally, user satisfaction scores in the intervention group surpassed those of the control group across all six dimensions and total satisfaction score (*P* < 0.001).

**Conclusions:**

Integrating management principles into HIS may provide an efficient and cost-effective potential for surgical specimen management in operating room, which aligned well with the current operational model that combined information system with manual specimen delivery process.

**Trial registration:**

Chinese Clinical Trial Registry ChiCTR2500114109, registered on 8 December 2025. This study was retrospectively registered.

**Supplementary Information:**

The online version contains supplementary material available at 10.1186/s12912-025-04288-y.

## Background

Surgical specimen examination serves as the ‘gold standard’ for disease diagnosis and treatment. The accuracy, safety and timeliness of specimen handling in the pre-analytical phase are essential to ensure the reliability of pathological diagnosis and the efficiency of surgical treatment [1]. The pre-analytical phase of surgical specimen, spanning from specimen collection to pathology department receipt, involves multiple personnel including the surgeons, operating room nurses, transport workers and pathology staff. Any error at any step, such as misidentification, mishandling, delays or missing may lead to severe patient consequences such as diagnostic errors, inappropriate treatment, repeat surgeries, physical and psychological trauma and increased healthcare costs [2, 3]. As early as 2007, errors in handling surgical specimen had drawn the attention of researchers at the Johns Hopkins University School of Medicine [4]. Studies reported that approximately 17% of specimen identification errors resulted in incorrect treatment, while nearly 6% led to adverse events [5]. Additionally, identifying and correcting such errors consumes significant time and resources, prolonging specimen handling and influencing surgical efficiency [6]. In recent years, researchers have continuously focused on reducing specimen handling errors and optimizing management protocols, making these areas focal points for perioperative staff.

Researches on improving specimen management quality in hospitals can be categorized into two strategies: one focuses on improving management methodology, while the other emphasizes upgrading equipment automation. As for management methodology study, D’Angelo R et al. [7] reduced specimen labeling errors by developing standardized procedures. A dental hospital in UK achieved sustained reduction of adverse events through regular staff training and clarifying responsibilities [8]. Bixenstine PJ et al. [6] developed 12 methods of identifying specimen handling defects to minimize errors and enhance patient safety. Meanwhile, the implementation of quality improvement project [2] and quality management tools such as Root Cause Analysis [9] and Healthcare Failure Mode and Effect Analysis [10] had significantly reduced specimen error rates and improved specimen management quality. Studies on specimen management through hardware-based solutions predominantly focused on technologies including barcode systems [11, 12], Quick Response code (QR) scanning [13], radio frequency identification and Internet of Things [14]. An intelligent automated specimen transfer system had also been developed in China for the pre-analytical phase of specimens, which ensured the quality of surgical specimen submission and alleviated the work pressure of nurses [15]. However, developing fully automated specimen management system is technologically costly, therefore posing significant challenges for large-scale implementation [16]. The use of information systems to enhance management efficacy become a widely implemented strategy in surgical and nursing practices in recent years [17, 18]. The integration of management principles into HIS has been demonstrated to improve patient safety outcomes [19, 20]. Based on the current situation, the manual transportation mode supported by Hospital Information System (HIS) remains the predominant approach for surgical specimen submission. Therefore, integrating multidimensional management protocols into the HIS will be a quite practical and broadly applicable solution for enhancing specimen management quality. ‌However, research on such integrated approaches of improving specimen management quality has rarely been reported.

The concept of chain management that focused on multi-step and cross-departmental coordination issues had been proven to be an effective method in improving nursing management quality [21, 22]. Therefore, based on the principle of chain management to analyze the high-risk steps within each phase of surgical specimen handling and the main problems in the inter-phase coordination steps, then embedding management protocols into the HIS is expected to significantly improve the quality of specimen management. In this study, we developed a ‘Surgical Specimen Chain Management Information System (SSCMIS)’ in a tertiary hospital in Zhejiang Province in China. SSCMIS matched the existing manual transportation mode and solved the main problems in the specimen management process, significantly reduced the unqualified rate of specimen handling, the duration of specimen handling and improved the satisfaction of medical staff.

## Methods

### Core concept of SSCMIS

**Fig. 1:**
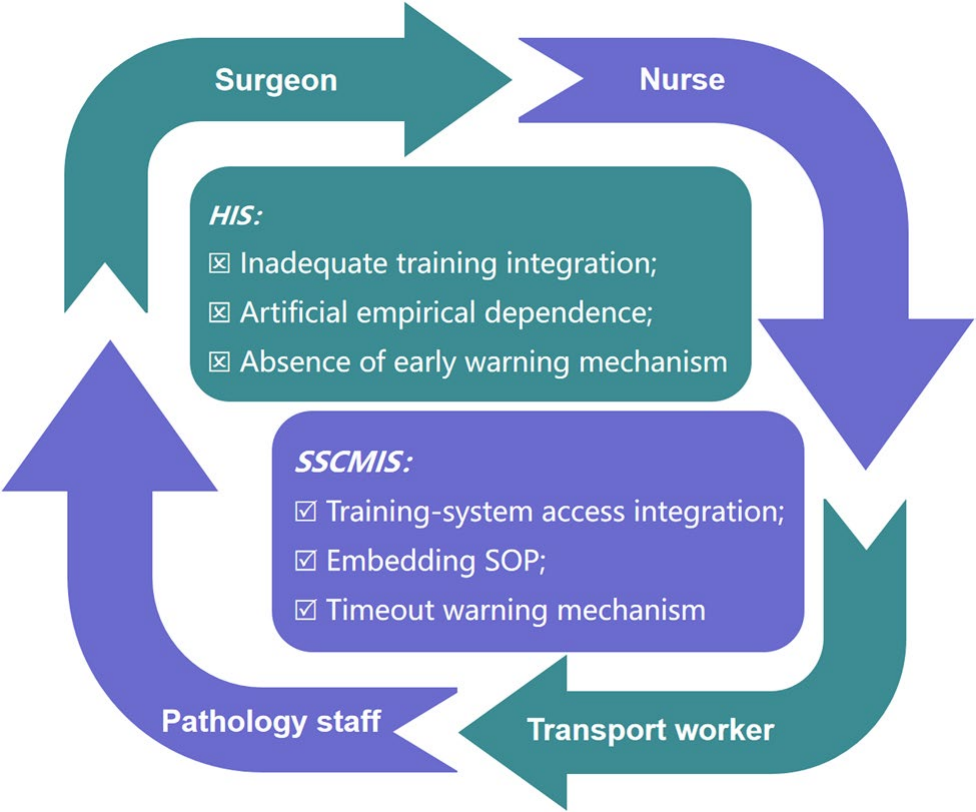
Improvement in SSCMIS compared with HIS for specimen handling

Based on the principle of chain management, in-depth analysis was conducted on the problems that are prone to occur during specimen handling, such as mislabeling, inadequate fixation, incorrect storage status (positioning and orientation), specimen retention and missing. It was found that the causes of the above problems lay in the following deficiencies of the existing HIS. (1) Irregular training integration. The accuracy and timeliness of specimen management rely on correct handling by personnel at each workflow stage. Current training methods rely on one-time verbal instruction with no follow-up assessments or refresher programs. Training content is not integrated with the HIS, exacerbating the administrative burden of manual training and evaluation. (2) Artificial empirical dependence. The current HIS lacks Standard Operating Procedure (SOP) of specimen handling and systemic safeguards‌ across multiple stages, from specimen collection to pathology department receipt. The quality of critical steps in specimen handling remains heavily dependent on individual competency. (3) Absence of early warning mechanism. Delayed handling or inadequate fixation often cannot be detected due to the failure of system for providing timely alerts to personnel. The existing HIS lacks timely reminders for operators and warning mechanisms for specimen handling timeout, resulting in the systemic failure to address delays in specimen handling. These three deficiencies of HIS are summarized in Fig. 1.

Regarding the above three issues, the SSCMIS can be modified accordingly. (1) Training-system access integration. The integration of staff regular training and assessment into system usage permissions ensures enhancement in training efficacy. (2) Embedding SOP. SOP of specimen handling through systematic operational checklists are embedded within the information system. Before performing the electronic signature, medical staff are required to check against the system prompts to prevent errors caused by human operation. (3) Timeout warning mechanism. Real-time monitoring protocol is used in the system. If the key step exceeds its designated timeframe, SSCMIS automatically notifies the circulating nurse, mitigating risks of specimen delays or undetected quality issues. The core concept is shown in Fig. 1. The detailed implementation methods of SSCMIS are described as follows.

### Aim

This study aimed to construct a chain management information system for surgical specimen, integrating management principles into the HIS, with the target of enhancing specimen handling qualified rate and improving operating room efficiency.

### Materials

The single-center, a pilot Quasi-Experimental study was conducted in the operating room of a tertiary hospital in Zhejiang Province, China between June 2024 and June 2025. The development and debugging of the SSCMIS took 9 months (June 2024 to February 2025). A convenient sample of 3,524 surgical specimens from the operating room between March to June 2025 were included as study subjects, comprising 2,518 permanent specimens (Permanent specimens were formalin-fixed and paraffin-embedded (FFPE)) and 1,006 intraoperative frozen specimens. Among these specimens, 1,780 specimens collected from March to April 2025 (1,259 permanent specimens and 521 intraoperative frozen specimens) were designated as the control group, while 1,744 specimens collected from May 2025 to June 2025 (1,259 permanent specimens and 485 intraoperative frozen specimens) formed the intervention group. The inclusion criteria included specimens requiring pathological diagnosis from elective and emergency surgeries in the following surgical departments: Neurosurgery, Head and Neck Thyroid Surgery, Breast Surgery, Thoracic Surgery, Gastrointestinal Pancreatic Surgery, Hepatobiliary Pancreatic Surgery, Anorectal Surgery, Gynecology, and Urology, with no statistically difference between two groups (Table 1). The cytology specimens were excluded.

#### Sample size

The sample size for comparing two proportions was calculated using the formula:

$$\:\mathrm{N}=\frac{{({\mathrm{U}}_{{\upalpha\:}}+{\mathrm{U}}_{{\upbeta\:}})}^{2}\:\times\:2\mathrm{P}(1-\mathrm{P})}{{({\mathrm{P}}_{1}-{\mathrm{P}}_{2})}^{2}}$$

where α = 0.05 and β = 0.1 [23]. From statistical tables, U_α_= 1.64 and U_β_= 1.28. The sample size calculation was based on previous study by Zhang et al. The qualified rate of permanent specimen was 97.6% in the control group and 99.2% in the intervention group in Zhang et al. [22]. The calculated sample size of permanent specimens for each group was N_1_ = N_2_ = 1049. Considering a 20% dropout rate, the sample size was increased to 1,259 cases for each group.

**Fig. 2:**
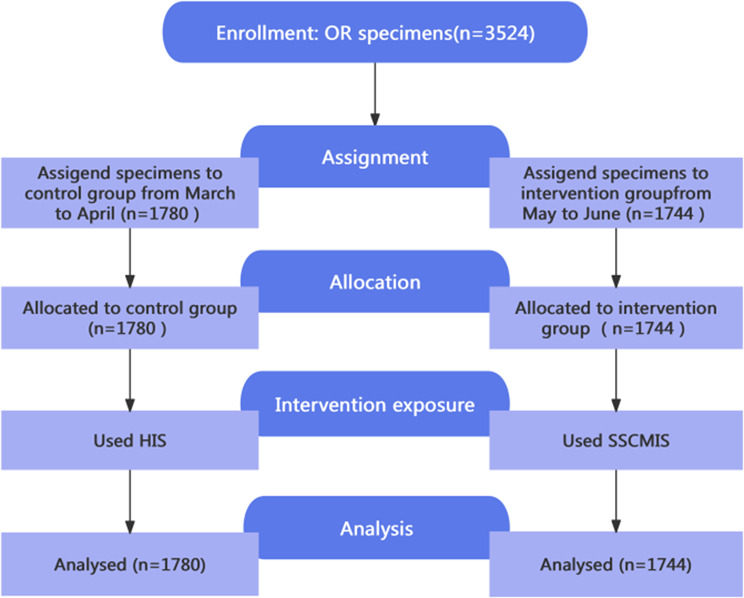
The TREND flow diagram of participants enrollment, assignment, allocation, intervention exposure and analysis in the Quasi-Experimental study

This study was designed and reported in accordance with the Transparent Reporting of Evaluations with Nonrandomized Designs (TREND) statement guidelines. The flow of participants through each stage of the study was shown in Fig. 2.

### Intervention

#### The control group

**Fig. 3:**
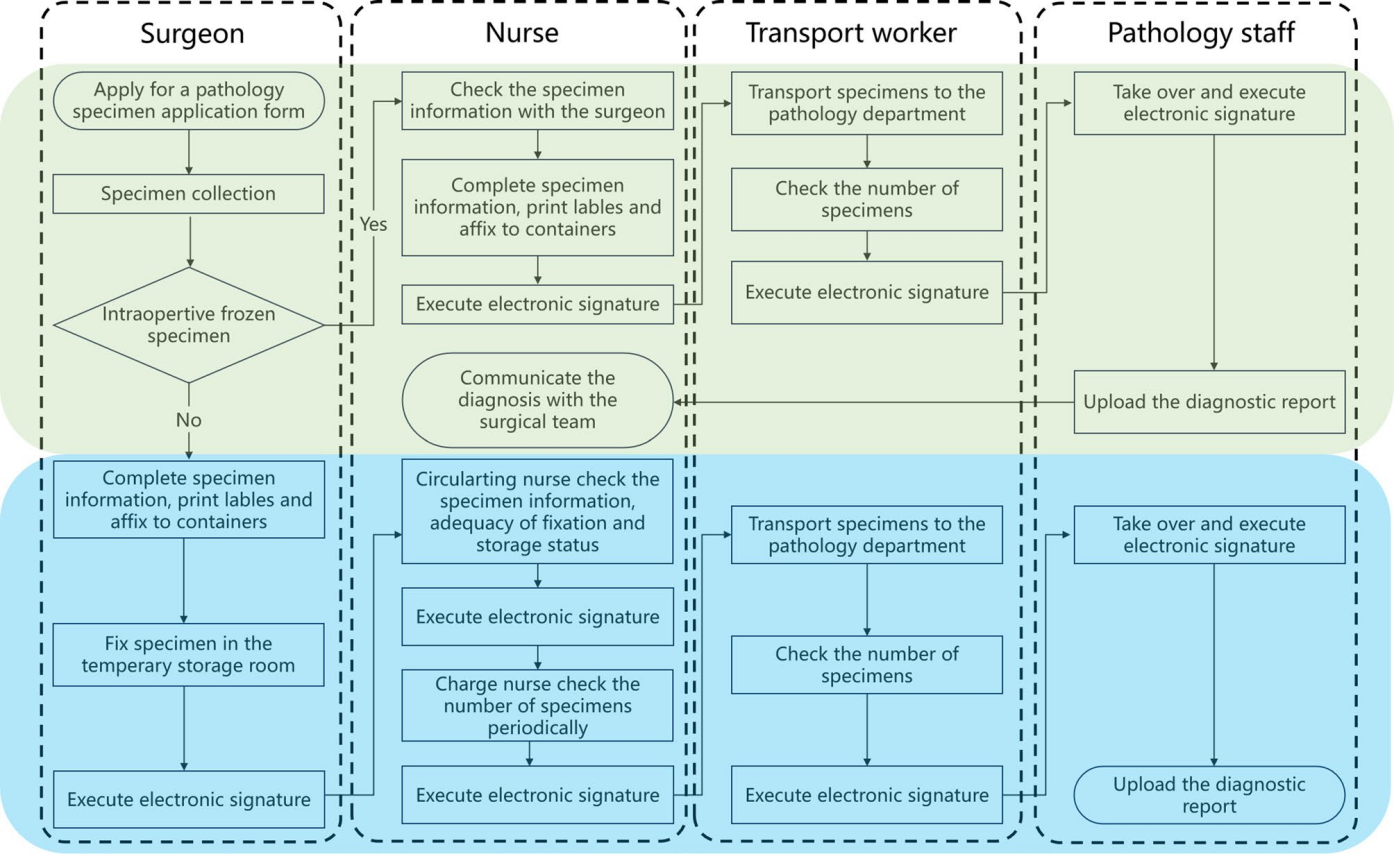
Surgical specimen submission process with blue shaded region for permanent specimen and green shaded region for intraoperative frozen specimen

The control group followed the current surgical specimen submission process (Fig. 3.) and used the HIS. HIS for specimen management was divided into two functional areas: permanent specimen submission and intraoperative frozen specimen submission. A pathology application form was issued before the surgery, the surgeon completed specimen information of the permanent specimen, printed the label and attached it to the specimen container after surgery. The surgeon then performed operations of specimen fixation and temporary storage, and electronic signature was required upon completion. The circulating nurse conducted regular checks on the specimen information, fixation quality and storage status of specimens. Electronic signature was applied after verification. The charge nurse periodically verified specimen quantities and executed electronic signature. Then, a transport worker delivered all specimens to the pathology department. Quantity verification was performed upon receipt along with electronic signature confirmation. The pathology staff completed receipt with e-signature and then processed specimen sectioning. Finally, the diagnosis results were published in HIS within 7 days.

Process for handling of intraoperative frozen specimen was as follows. Upon intraoperative frozen specimen collection was finished, the operating room nurse confirmed specimen information with the surgeon and completed the specimen information in HIS. Printed labels were attached to specimen containers, followed by electronic signature confirmation of the circulating nurse. Then the transport worker verified the specimen count and completed an electronic signature after the specimen was transported to the pathology department. The pathology department should upload the diagnostic report to HIS within 30 min after completing an electronic signature for receipt. At the same time, the circulating nurse should immediately communicate the diagnosis to the surgical team.

#### The intervention group

The intervention group was implemented with SSCMIS for specimen handling based on the framework of control group. The SSCMIS used in intervention group specifically strengthened management of operational procedure training and high-risk procedures in specimen handling, adhering to the three core principles showed in Fig. 1.

##### Establishing a multidisciplinary team

A multidisciplinary team‌ was established for surgical specimen management, consisting of two head nurses, one charge nurse, one core nurse, one pathology personnel and one information technology (IT) specialist. The head nurses were responsible for coordinating and promoting the construction and implementation of the system. The charge nurse and core nurse were responsible for training on the use of SSCMIS and data collection. The pathology personnel conducted training for pathology staff on standardized surgical specimen reception. The IT specialist developed, debugged and maintained the SSCMIS, ensuring its stable operation.

##### Training for SSCMIS

Training responsibilities were allocated as follows. The charge nurse was responsible for training the operating room nurses. The core nurse was in charge of training transport workers, and the assigned circulating nurse undertook surgeons’ instruction. They were trained primarily to learn the functions and usage of the system, as well as high-risk procedures in specimen delivery. Only the staff who had successfully handled the specimen through the SSCMIS was authorized to use it formally.

##### Operating environment of SSCMIS

The SSCMIS was integrated into HIS in a modular format and operated on a mobile device. Operators can access this function by logging in the mobile device with their personal identity number (ID) and password.

##### System functional modules

**Fig. 4:**
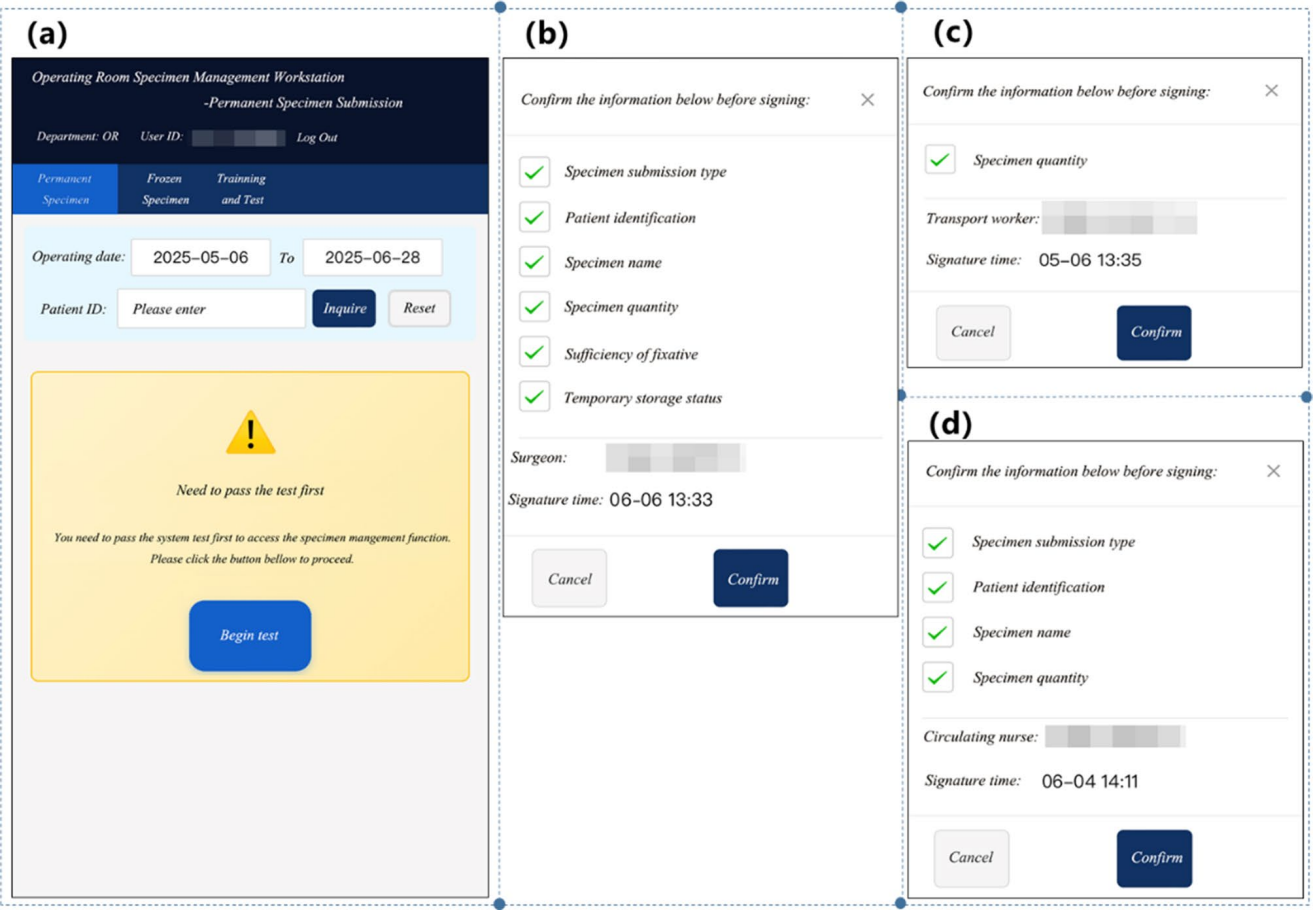
Interface of SSCMIS. Staff training and assessment module (**a**). SOP prompt for surgeon (**b**) and transporter worker (**c**) of permanent specimen. SOP prompt for OR circulating nurse of intraoperative frozen specimen (**d**)

The system consisted of two functional modules. The first module was staff training and assessment. This module evaluated personnel through customized tests specific to their roles based on specimen management regulation. After logging in the SSCMIS, system identified the role of users (e.g., surgeon, operating room nurse or transport worker) and checked the time elapsed since their last test. If two months had passed since their last test, the system automatically activated a new assessment interface (as shown in Fig. 4(a)). And the staff must pass the test to retain authorization for surgical specimen management operations.

The second module of SSCMIS was operation. The same as HIS, operation module of SSCMIS was divided into two types: permanent specimen submission and intraoperative frozen specimen submission, allowing the surgeons and operating room nurses to perform functions such as order inquiry, application, specimen information printing, submission, tracking, and diagnosis inquiry for the two types of surgical specimen through the patient’s ID. Design of the operational modules adhered to two core concepts: embedding SOP and timeout warning mechanism.

With SOP embedded in SSCMIS, staff were required to verify key steps based on system prompts before executing electronic signatures. As for permanent specimen, it went through multiple steps from specimen collection to pathological receipt. Surgeons should verify submission type, patient identification, specimen name, specimen quantity, sufficiency of fixative and correctness of temporary storage status (Fig. 4(b)). In order to ensure correctness once again, prompt popped up in the SSCMIS for circulating nurse was the same as surgeon’s. While for charge nurse and transport worker, confirming consistency between actual specimen count and system-recorded quantity would be prompted by the SSCMIS (Fig. 4(c)). For the intraoperative frozen specimen, the circulating nurse must confirm specimen submission type, patient identification, specimen name and quantity (Fig. 4(d)). The information that the transport worker should verify was the same as the permanent specimen’s.

**Fig. 5:**
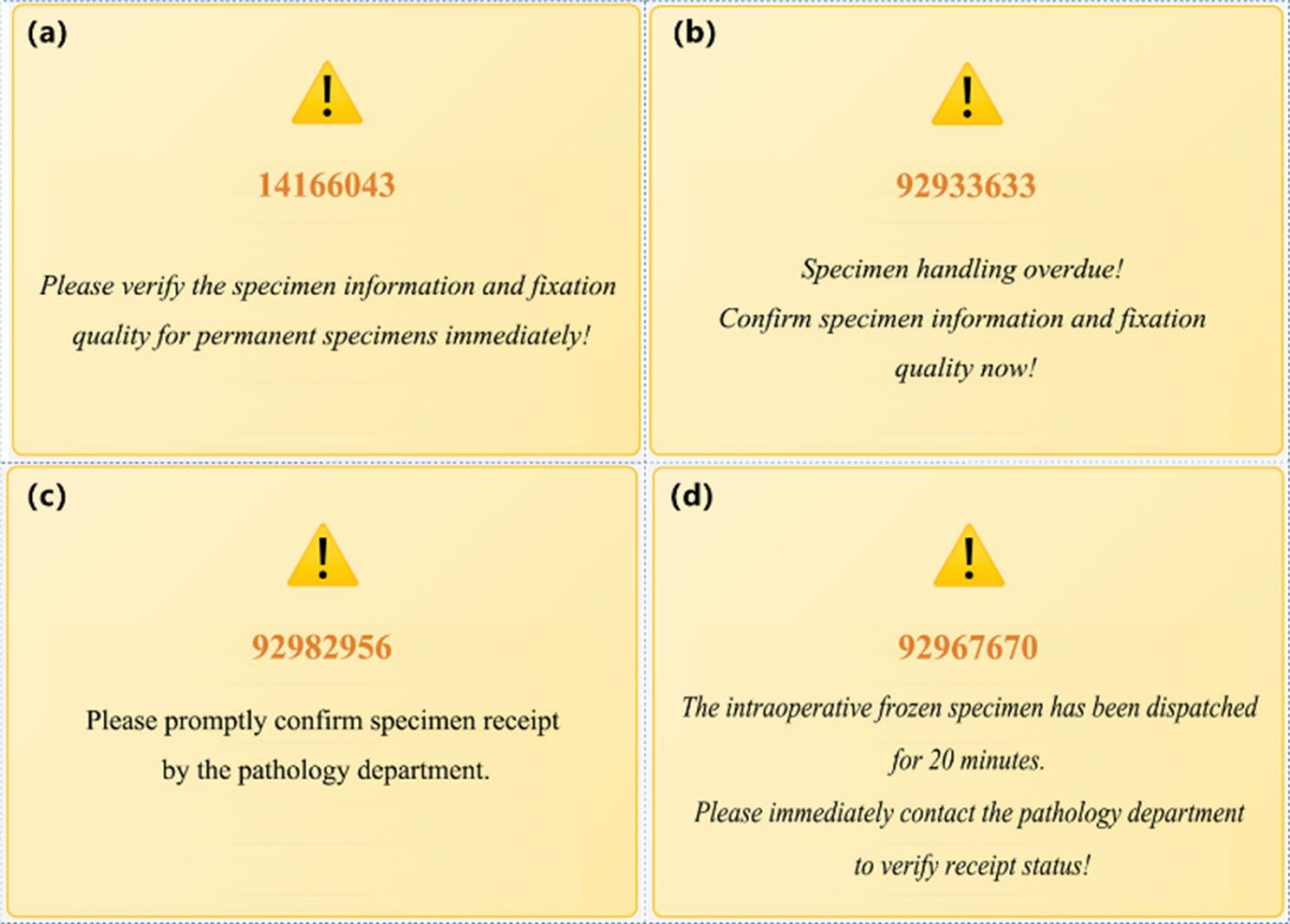
Interface of the preset timeframe reached 80% (**a**) and 100% (**b**) for permanent specimen. Interface of the preset timeframe reached 80% (**c**) and 100% (**d**) for intraoperative frozen specimen

Besides embedding SOP, time-out warning mechanism was another core concept of SSCMIS. Based on historical data from this hospital, SSCMIS established preset timeframes for critical steps in the specimen handling process. The high-risk phase for permanent specimen lay in the circulating nurse’s verification of specimen information and fixation quality after the surgeon temporarily stored the specimen. Given the widespread staff shortages in Chinese operating room and the critical impact of fixation quality for pathological diagnosis, the system mandated that circulating nurse verified permanent specimen within 60 min post-surgery to ensure correct specimen handling. At 80% of the allotted time (48 min), if the signature had not been performed, a system pop-up alert triggered ‘Please verify the specimen information and fixation quality for permanent specimens immediately!’ (Fig. 5(a)). If no electronic signature was recorded by the 60-minute deadline, an urgent alert (‘Specimen handling overdue! Confirm specimen information and fixation quality now!’) appeared to force the circulating nurse to complete verification immediately (Fig. 5(b)). For intraoperative frozen specimen, the total timeframe was set for 20 min from the circulating nurse’s signature to the pathology department’s confirmation signature. When the preset timeframe reached 80% (16 min) completion while the pathology department’s signature had not been performed, system pop-up alerts were triggered for the circulating nurse ‘Please promptly confirm specimen receipt by the pathology department.’ (Fig. 5(c)). If the pathology department’s electronic confirmation remained uncompleted by the 20-minute deadline, an urgent alert appeared ‘The intraoperative frozen specimen has been dispatched for 20 minutes. Please immediately contact the pathology department to verify receipt status!’ (Fig. 5(d)).

### Measurements

#### (1) Characteristics of specimen source (Appendix 1 questionnaire.pdf)

Specimen source encompassed two components: firstly, the surgical context (elective versus emergency surgeries), and secondly, the originating surgical departments.

#### (2) Specimen handling qualified rate (Appendix 1 questionnaire.pdf)

The qualified rates of specimen were categorized into two categories: the qualified rate of permanent specimen and the qualified rate of intraoperative frozen specimen. The rate was calculated as the ratio of qualified cases to the total specimen cases.

In this study, the qualification of permanent specimen was determined by five mandatory criteria, with failure to meet any single criterion resulting in disqualification of the specimen handling process.

- Accurate patient identification: correct patient name and ID.
- Accurate specimen name: correct anatomical site.
- Proper placement location: specimen must be located in the specific box labeled with the corresponding operating room number.
- Standard fixation: fixative must fully submerge the specimen, with a hermetical container to prevent leakage.
- Correct orientation: specimen container must be positioned vertically upright.

The qualified rate of intraoperative frozen specimen was determined by two mandatory criteria, with failure to meet any single criterion resulting in disqualification of the specimen handling process.

- Accurate patient identification: correct patient name and ID number.
- Accurate specimen name: correct anatomical site.

#### (3) Duration of specimen handling (Appendix 1 questionnaire.pdf)

‌ A comparative record was conducted between the two groups regarding the handling duration of the permanent specimens and the intraoperative frozen specimens. As for permanent specimen, the time was recorded from the surgery completion to verification and sign-off by the circulating nurse in the temporary storage room. While for intraoperative frozen specimen, duration from electronic signature by the circulating nurse to successful receipt confirmation by the pathology department was recorded.

#### (4) Satisfaction of the operating room nurses

Satisfaction was assessed using the Clinical Nursing Information System Effectiveness Evaluation Scale developed by Zhao Yongxin et al. [24] in 2020. This scale included ‌23 items across 5 dimensions‌, with a total Cronbach’s alpha of ‌0.768‌ and test-retest reliability of ‌0.849‌, demonstrating strong validity and reliability for evaluating nursing information systems.

### Data collection

Operating room nurses were required to document the characteristics of specimen source and all types of specimen handling errors into the designated Excel daily. The researcher conducted daily random sampling of 45 specimens (generating random numbers). The charge nurse and core nurse recorded the characteristics of specimen source, processing types of specimens and the types and numbers of unqualified cases, while also extracting the duration of specimen handling from the system. Data collection continued until the required sample size of permanent specimens for this study was achieved.

Satisfaction of the operating room nurses with the HIS and SSCMIS was evaluated via electronic questionnaire QR codes distributed by the research team. The survey was administered with pretest to posttest, and the responses constituted paired data. The satisfaction with HIS was assessed prior to SSCMIS implementation and the satisfaction with SSCMIS was assessed after completing the sample size of intervention specimens.

### Data analysis

A database was established using Excel with double data entry. Data analysis was performed in SPSS 25.0. Measurement data that conformed to a normal distribution were described as mean ± standard deviation and analyzed using t-test. Non-normally distributed measurement data were described as median (interquartile range) and analyzed via rank sum test (Mann-Whitney U test and Wilcoxon signed-rank test). Count data were summarized as frequency (percentage) and evaluated using Chi-square test. Statistical significance was defined as *P* < 0.05. The odds ratio (OR), the confidence interval (CI) for Chi-square test and rank-biserial correlation (R), CI for Mann-Whitney U test were determined to analyze the possible influence of the variables.

## Results

### Characteristics of specimen source

Table 1 showed there were no statistically significant differences in the source of specimens among the two groups, indicating that the two groups were comparable at important baseline characteristics (*P* > 0.05).

**Table 1:** Comparison of the characteristics of specimen source of two groups

The type of specimens	Item		Control group	Intervention group	χ^2^	*P*
			**Frequency**	**Frequency**		
The permanent specimen	Elective/Emergency surgeries		1130/129 89.8%/10.2%	1108/151 88%/12%	1.945	0.163
	The source of specimens	Neurosurgery	92(7.3%)	103(8.2%)	8.233	0.411
		Head and Neck Thyroid Surgery	149(11.8%)	154(12.2%)		
		Breast Surgery	84(6.7%)	78(6.2%)		
		Thoracic Surgery	142(11.3%)	151(12.0%)		
		Gastrointestinal Pancreatic Surgery	138(11.0%)	153(12.2%)		
		Hepatobiliary Pancreatic Surgery	126(10%)	132(10.5%)		
		Anorectal Surgery	142(11.3%)	138(11.0%)		
		Gynecology	157(12.5%)	169(13.4%)		
		Urology	229(18.2%)	181(14.4%)		
The intraoperative frozen specimen	Elective/Emergency surgeries		498/23 95.6%/4.4%	471/14 97.1%/2.9%	1.655	0.198
	The source of specimens	Neurosurgery	12(2.3%)	9(1.9%)	7.229	0.512
		Head and Neck Thyroid Surgery	107(20.5%)	94(19.4%)		
		Breast Surgery	151(29%)	114(23.5%)		
		Thoracic Surgery	58(11.1%)	67(13.8%)		
		Gastrointestinal Pancreatic Surgery	48(9.2%)	54(11.1%)		
		Hepatobiliary Pancreatic Surgery	56(10.7%)	62(12.8%)		
		Anorectal Surgery	21(4.0%)	18(3.7%)		
		Gynecology	48(9.2%)	51(10.5%)		
		Urology	20(3.8%)	16(3.3%)		

### The handling qualified rate of permanent specimen and intraoperative frozen specimen

After the implementation of SSCMIS, the intervention group demonstrated a higher qualified rate of permanent specimen compared to the control group in terms of fixative adequacy(*p* = 0.006, OR = 3.371, 95% CI: 1.349–8.422), qualification of orientation(*P* = 0.045, OR = 3.019, 95% CI: 0.971–9.387), and total qualified specimen handling(*P* < 0.001, OR = 3.799, 95% CI: 2.048–7.047), with a statistically significant difference and a moderate to large effect sizes except qualification of orientation. As for intraoperative frozen specimen, there was no statistically significant difference in the handling qualified rate between the two groups (*P* > 0.05), as detailed in Table 2.

**Table 2:** Comparison of the handling qualified rate of the permanent specimen and intraoperative frozen specimen of two groups

The type of specimens	Item	Control group	Intervention group	χ^2^	*P*	OR (95% CI)
		**Frequency**	**Frequency**			
The permanent specimen	Patient identification Right/Wrong	1257/2 99.8%/0.2%	1259/0 100%/0%	/	0.5*	/
	Specimen name Right/Wrong	1253/6 99.5%/0.5%	1258/1 99.9%/0.1%	/	0.124*	6.024/(0.724–50.108)
	Placement location Right/Wrong	1251/8 99.4%/0.6%	1257/2 99.8%/0.2%	3.614	0.057	4.019(0.852–18.964)
	Fixative adequacy/inadequacy	1239/20 98.4%/1.6%	1253/6 99.5%/0.5%	7.617	0.006	3.371(1.349–8.422)
	Orientation Right/Wrong	1247/12 99.0%/1%	1255/4 99.7%/0.3%	4.026	0.045	3.019(0.971–9.387)
	total Specimens handling ​Qualified/Unqualified	1211/48 96.2%/3.8%	1246/13 99.0%/1.0%	20.581	< 0.001	3.799(2.048–7.047)
The intraoperative frozen specimen	Patient identification Right/Wrong	520/1 99.8%/0.2%	485/0 100%/0%	/	1*	/
	Specimen information Right/Wrong	519/2 99.6%/0.4%	485/0 100%/0%	/	0.5*	/

* Fisher’s exact test

### Duration of specimen handling

As shown in Table 3, the duration of specimen handling of both permanent specimen and intraoperative frozen specimen in the intervention group were significantly shorter than those in the control group, and the differences were statistically significant (*P* < 0.001), with a large effect size of permanent specimen (*R* = 0.59) and a medium to large effect size (*R* = 0.47) of intraoperative frozen specimen.

**Table 3:** Comparison of specimen handling duration of permanent specimen and intraoperative frozen specimen of two groups

The type of specimens	Control group	Intervention group	Mann-Whitney U test		*R* (95% CI)
	Median (P25, P75) (minutes)	Median (P25, P75) (minutes)	Z	*P*	
Duration of permanent specimen	25(20, 29)	17(13, 21)	-25.820	< 0.001	0.59(0.56–0.62)
Duration of intraoperative frozen specimen	6(5, 7)	5(4, 5)	-13.150	< 0.001	0.47(0.41–0.52)

### Satisfaction of the operating room nurses

A total of 76 valid responses were collected from operating room nurses regarding their satisfaction with SSCMIS and HIS. The results showed that compared with HIS, SSCMIS achieved significantly higher scores across all six dimensions (system quality, information quality, service quality, user satisfaction, net benefits) and total satisfaction score (*P* < 0.001), as detailed in Table 4.

**Table 4:** Comparison of satisfaction with SSCMIS and HIS of the operating room nurses

Item	Control group	Intervention group	Wilcoxon Signed Rank Test	
	Median (P25, P75)/ Average ± standard deviation*	Median (P25, P75)	Z	*P*
System quality	18(17.25, 19)	19 (18, 19)	-4.883	< 0.001
Information quality	20 (19, 21.75)	22 (21, 23)	-6.977	< 0.001
Service quality	16 (15, 17)	17 (16, 18)	-3.743	< 0.001
User satisfaction	21 (20, 22)	23 (21, 24)	-6.473	< 0.001
Net benefits	13 (12, 14)	21 (19, 21.75)	-7.612	< 0.001
Total satisfaction score	87.83 ± 3.20	100.5(99, 102.75)	-7.584	< 0.001

* Median (P25, P75): The description for non-normally distributed data

Average ± standard deviation: The description for normally distributed data

## Discussion

Surgical specimen management involves multi-departmental coordination across multiple workflow stages. Ensuring seamless execution at each stage is critical for improving the specimen handling quality during the pre-analytical phase. Therefore, strengthening staff training and standardizing protocols remain vital strategies for enhancing specimen quality [25]. The absence of a timeout alert in HIS, combined with high clinical workload, increases the risk of specimen missing or delayed specimen verification by circulating nurse. Meanwhile, current training relies on manual methods lacking system interactivity. To overcome these limitations within the prevailing status of combination information system with manual handling, the SSCMIS in this study developed three key innovations. First of all, periodic training and competency assessments tailored to different roles were implemented in SSCMIS, enhancing training effectiveness across all personnel and ensuring seamless execution at each workflow stage. Additionally, SOP in SSCMIS before signature at each phase, effectively reducing errors such as specimen misidentification and inadequate fixation. Integrated with timeout alert function of SSCMIS, this approach enabled staff to detect and resolve specimen missing and unqualified issues, ensuring smooth interdepartmental coordination. Consequently, the handling qualified rate of permanent specimen in fixative adequacy (*p* = 0.006, OR = 3.371, 95% CI: 1.349–8.422) and total qualified specimen handling (*P* < 0.001, OR = 3.799, 95% CI: 2.048–7.047) significantly improved, indicating that the odds of specimen handling unqualified rate were approximately in excess of threefold higher in the control group than in the intervention group, which could further ensuring diagnostic precision in pathological analysis [26]. The qualification of orientation of permanent specimen in the two groups showed a statistically significant difference (*P* = 0.045), but the association had limited precision (the OR confidence interval including 1). This suggests that the finding is noteworthy but preliminary, and future studies with larger sample sizes are needed to provide more precise estimate. The absence of significant differences in patient identification, specimen name and placement location between the two groups may be attributed to enhanced awareness among medical staff and the limited sample size in the current study. Wu et al. [19] demonstrated that integrating Heinrich’s Law principles with mobile information system significantly enhanced patient safety during the perioperative period. Similarly, Li et al. [20] standardized nursing risk assessment and management protocols by developing a nursing information system, which increased the implementation rate of nursing risk evaluations and improved safety outcomes. These findings aligned closely with the conclusion of this study that combining management principles with information system elevated overall quality control.

This study also showed that there were no statistically significant differences in the handling qualified rate of intraoperative frozen specimen between two groups. It may be due to the fact that the sample size frozen specimens was actually limited. Additionally, the submission process of intraoperative frozen specimen was less complex than that of permanent specimen, which may also induce the SSCMIS’s impact was less pronounced, indicating the limitation of the system.

Results of this study demonstrated that with the usage of SSCMIS, duration of specimen handling including both permanent specimen (*P* < 0.001, *R* = 0.59, 95% CI: 0.56–0.62) and intraoperative frozen specimen (*P* < 0.001, *R* = 0.47, 95% CI: 0.41–0.52) in the intervention group were shorter than those in the control group. They were due to two reasons. The timely reminder and timeout warning for specimen handling ensured that staff could complete specimen handling within the specified time, directly shortening the specimen handling time. The second reason was that the qualified rate of specimen handling had increased, and the number of cases in which medical staff correct unqualified specimen had decreased. The magnitude of effect could translate into saving an average of 8 min per permanent specimen and 1 min per intraoperative frozen specimen in the intervention group. Therefore, in the long run, it can be expected that with the use of SSCMIS, the standardization of specimen handling and the effectiveness of personnel training would be improved. Whether it was permanent specimen or intraoperative frozen specimen, the handling time of specimen would decrease with the increase of specimen handling qualified rate, which could significantly improve operational efficiency.

The development of SSCMIS had led to a significant improvement in users’ satisfaction (*P* < 0.001). On one hand, since the marked improvement in specimen handling qualified rate reduced the frequency and time that operating room nurses spent on correcting errors, thereby alleviating clinical workloads and boosting nurse satisfaction. On the other hand, as a critical determinant of satisfaction, user acceptance was influenced by technology readiness and system quality [27]. Before this study, the medical staff had been familiar with the HIS, in addition the pre-implementation training for SSCMIS users significantly boosted acceptance of the new system, establishing a critical prerequisite for successful adoption of SSCMIS [28]. Optimized the existing system, SSCMIS was less difficult for users to operate and aligned closely with user needs, which resulted in high satisfaction. These outcomes validated its efficacy as a successful informatics solution [29].

The SSCMIS might demonstrate cost-effectiveness in two key areas. Regarding IT development, the SSCMIS required relatively minimal development effort, as it interfaced with the existing HIS for specimen management, as opposed to building an entirely new system. Furthermore, the associated technical costs were lower than those for implementing technologies such as the Internet of Things or intelligent automated systems [16]. In terms of training time, its HIS-based interface ensured a steep learning curve, with our experience showing proficiency was achieved within 1–2 h. Therefore, it is reasonable to speculate that SSCMIS holds a positive cost-effective prospect.

## Limitations

It should be acknowledged that this study had certain limitations that may have influenced the reported results. Firstly, this study was conducted in only one tertiary hospital in China, which may limit the generalizability of its findings to other medical institutions. Secondly, due to constraints in the study design, a potential risk of bias could not be entirely excluded. The convenience sampling method employed in this study had methodological limitations and was suitable for the pilot phase of this study. Furthermore, as the control group was implemented earlier than the intervention group, the time effect might also have influenced the results. Although the study yielded promising findings, larger-scale and longer-term studies in multicenter areas will be warranted to explore the effects of the SSCMIS.

## Conclusion

Based on the chain management mechanism, SSCMIS have significantly improved the qualified rate of specimen handling and reduced the time spent by medical staff on specimen handling, thereby greatly enhancing the satisfaction of operating room nurses and boosting the efficiency of operating room management. Integrating management methodologies into the HIS may provide an efficient and cost-effective potential for surgical specimen management, which closely aligns with the current operational model that combines digital management with manual specimen delivery process.

## Supplementary Information

Below is the link to the electronic supplementary material.

Supplementary Material 1

## Data Availability

The datasets used and analyzed during this study are available from the corresponding author upon reasonable request.

## Abbreviations

QR: Quick Response
HIS: Hospital Information System
SSCMIS: Surgical Specimen Chain Management Information System
SOP: Standard Operating Procedure
FFPE: Formalin-Fixed and Paraffin-Embedded
IT: Information Technology
ID: Identity number
OR: Odds ratio
CI: Confidence interval
R: Rank-biserial correlation
