# Supplementary material for: Effect of Surgical Specimen Chain Management Information System on specimen management in operating room: a pilot Quasi-Experimental study
Source: BMC Nurs. 2026 Jan 6;25:114. doi: 10.1186/s12912-025-04288-y (PMC12870954; doi:10.1186/s12912-025-04288-y)
Supplement: Supplementary file 1 — Supplementary Material 1 [file 12912_2025_4288_MOESM1_ESM.pdf]

Specimen Management Information Registration

Serial No.	Specimen Type (Permanent/ Frozen)	Department	Surgery Type (Elective/ Emergency)	Specimen Handling (Correctness/Error)		Permanent Specimen		Intraoperative Frozen Specimen	
				Correctness （Please mark √）	Type of Specimen Handling Error	Time of Surgery Completion	Time of Circulating Nurse's Signature	Time of Circulating Nurse's Signature	Time of Pathology Department Reception
